# Integration of Terrestrial Laser Scanning and NURBS Modeling for the Deformation Monitoring of an Earth-Rock Dam

**DOI:** 10.3390/s19010022

**Published:** 2018-12-21

**Authors:** Hao Xu, Haibo Li, Xingguo Yang, Shunchao Qi, Jiawen Zhou

**Affiliations:** 1 State Key Laboratory of Hydraulics and Mountain River Engineering, Sichuan University, Chengdu 610065, China; 2017223060078@stu.scu.edu.cn (H.X.); shunchaoqi@scu.edu.cn (S.Q.); 2 College of Water Resource and Hydropower, Sichuan University, Chengdu 610065, China; hbli@stu.scu.edu.cn (H.L.); 89022251@163.com (X.Y.)

**Keywords:** earth-rock dam, 3D visualization, deformation monitoring, terrestrial laser scanning (TLS), NURBS

## Abstract

A complete picture of the deformation characteristics (distribution and evolution) of the geotechnical infrastructures serves as superior information for understanding their potential instability mechanism. How to monitor more completely and accurately the deformation of these infrastructures (either artificial or natural) in the field expediently and roundly remains a scientific topic. The conventional deformation monitoring methods are mostly carried out at a limited number of discrete points and cannot acquire the deformation data of the whole structure. In this paper, a new monitoring methodology of dam deformation and associated results interpretation is presented by taking the advantages of the terrestrial laser scanning (TLS), which, in contrast with most of the conventional methods, is capable of capturing the geometric information at a huge amount of points over an object in a relatively fast manner. By employing the non-uniform rational B-splines (NURBS) technology, the high spatial resolution models of the monitored geotechnical objects can be created with sufficient accuracy based on these point cloud data obtained from application of the TLS. Finally, the characteristics of deformation, to which the geotechnical infrastructures have been subjected, are interpreted more completely according to the models created based on a series of consecutive monitoring exercises at different times. The present methodology is applied to the Changheba earth-rock dam, which allows the visualization of deformation over the entire dam during different periods. Results from analysis of the surface deformation distribution show that the surface deformations in the middle are generally larger than those on both sides near the bank, and the deformations increase with the increase of the elevations. The results from the present application highlight that the adhibition of the TLS and NURBS technology permits a better understanding of deformation behavior of geotechnical objects of large size in the field.

## 1. Introduction

The deformation distribution and evolution are present as important indications of the instability of large artificial and natural structures such as tunnels, bridges, and landslide [1,2,3,4]. Characterizing the in-situ deformation behavior of huge/important structures helps to understand the underlying mechanism of sliding and to predict the possibilities of catastrophic collapse. Thus, periodic monitoring of deformation of the structure has been an important task over the whole lifecycle of infrastructures of great importance, e.g., for ensuring their safety during construction as well as for their post-construction maintenances. Take the example of a dam: many monitoring instruments have been adopted with proper methodologies to appraise the condition and safety of the dam over the past decades [5,6,7]. Using traditional methods are possible for monitoring over a relatively large area. However, one of the common features of all these approaches e.g. general geodesy method and GPS static method, is that they can only provide point-wise information. For the dams with large scale, the number of the monitoring points is usually rather limited in terms of sufficiently characterizing the deformation characteristics. Synthetic Aperture Radar (SAR) is a relatively new technology, whose variant, GB-SAR, is used for the deformation measurement and monitoring of dam [8,9,10]. It needs to be recognized that SAR offers high sensitivity to small displacements. The most advanced GB-SAR is even capable of providing millimeter precision. Conversely, the main shortcoming of SAR is that it can only allow for deformation detection along the sensors-target line of sight. Furthermore, the data processing and analysis tools of SAR are quite complex, which makes it difficult to be applied into practice. 

In contrast, the terrestrial laser scanning has a definite advantage of providing point clouds composed of millions of points with high accuracy and high spatial resolution, and the point clouds can be used to detect vertical and horizontal deformation through relatively simple process. Nevertheless, the point cloud data acquired from the laser scanner cannot be processed in the same way as that for the data from the traditional methods, since the laser pulse emitted from the machine is not necessarily aimed at the same place of the target object in different scanning operations [11]. Additionally, the point density of the target object varies in different measurement campaigns, which making direct comparison between points cloud obtained sequentially is not favorable. 

Hence, how to extract information of the dam deformation from the terrestrial laser scanning (TLS) data is a challenging and important task, which is the focus of many recent works carried out on the deformation detection by the application of TSL [12,13,14,15,16]. A simple means of extracting the deformation is to compare the point-cloud gathered in different epochs [17,18,19]. This, however, can only show the general deformation tendency exhibited by the target object, which implies that it only allows for a qualitative analysis of the deformation rather than a quantitative analysis. The traditional method for surface reconstruction is point cloud gridding. A typical type of gridding algorithm, called the Delaunay triangulation algorithm, is widely used in modern programs, like Rapid Form and Riscan Pro. Due to its flexibility and adaptability, the Delaunay triangulation algorithm is widely accepted for reconstructing the surface of the objects with irregular shape, such as land relief and mechanical parts [20,21,22]. Unfortunately, the accuracy of the triangulated irregular network model is not satisfactory, making the subsequent deformation detection difficult to accomplish. Besides, the triangulation algorithm is rather disappointing in the aspect of noise reduction. 

In this paper, a detailed deformation measurement method, based on combination of TLS and NURBS technologies, is presented. The method is established on the basis of the quadrangular surface domain parameters for deformation monitoring. By specifying the quadrangular surface domain parameters, the four corner points of the NURBS fitting surface are exactly positioned at the four vertexes of the control mesh. Thus, the precision of the NURBS fitting surface reaches up to a couple of millimeters and is higher than the traditional NURBS surface. The performance of this present method in the field is illustrated by an application to a selected hydraulic structure, the Changheba Dam, located in Southwest China. To acquire the detailed and accurate 3D data rapidly and efficiently, the TLS technology is first applied to take inventories. To fully take advantage of the point cloud data, the digital surface model of the Changheba Dam with high accuracy and high spatial resolution is established by using the NURBS surface modeling technology. Through the comparison between multi-temporal models, the deformation characteristics of the Changheba Dam in different stages are analyzed. The results presented in this study illustrate the applicability of the present methodology to the precise deformation monitoring over large regions. 

## 2. Background

### 2.1. The Changheba Dam

**Figure 1:**
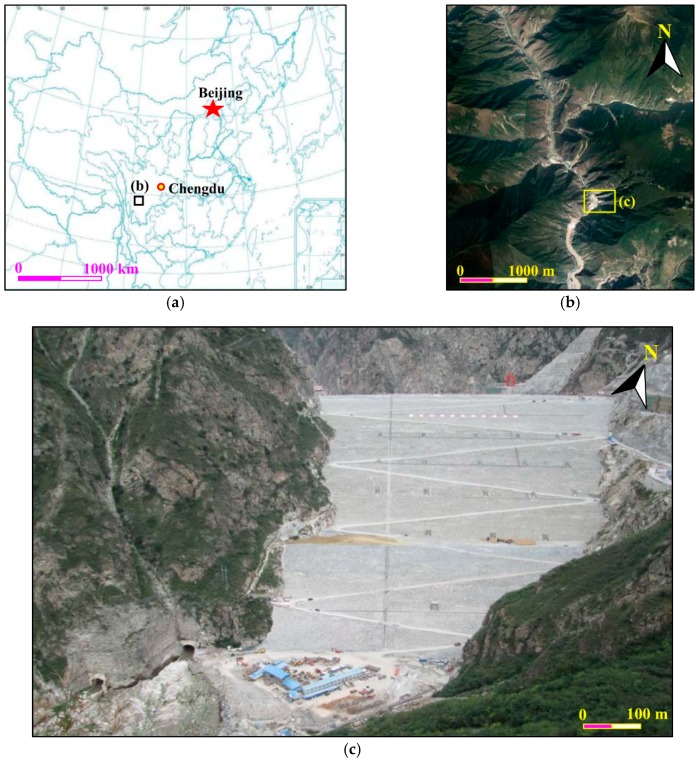
Location and layout of the dam at Changheba Hydropower Station: (**a**) and (**b**) location of the Changheba Hydropower Station, (**c**) layout of the dam at Changheba Hydropower Station.

Located about 360 km southwest of Chengdu, the capital city of Sichuan province in Southwest China, the Changheba Hydropower Station retains the Dadu River over a basin area of 56,648 km^2^ (Figure 1a,b). The hydro-technical system, with the primary purpose of exploiting the hydropower potential of the Dadu River, consists of an earth-rock dam, a spillway system and a water diversion and power generation facilities. The construction of the whole project was completed in April, 2018. With the maximum height of 240.0 m, the Changheba dam is the highest earth-rock dam built with gravel and soil core wall in China at present (Figure 1c). The length and width of the dam crest are 502.8 m and 16.6 m, respectively. Its gross filling volume is about 3.42 × 10^7^ m^3^, the largest volume of core wall earth-rock dam constructed in China. The dam can be divided into eight different zones according to the filling materials. The distinct mechanical properties of the filling materials make the deformation of the dam very intricate.

### 2.2. Geodetic Network

**Figure 2:**
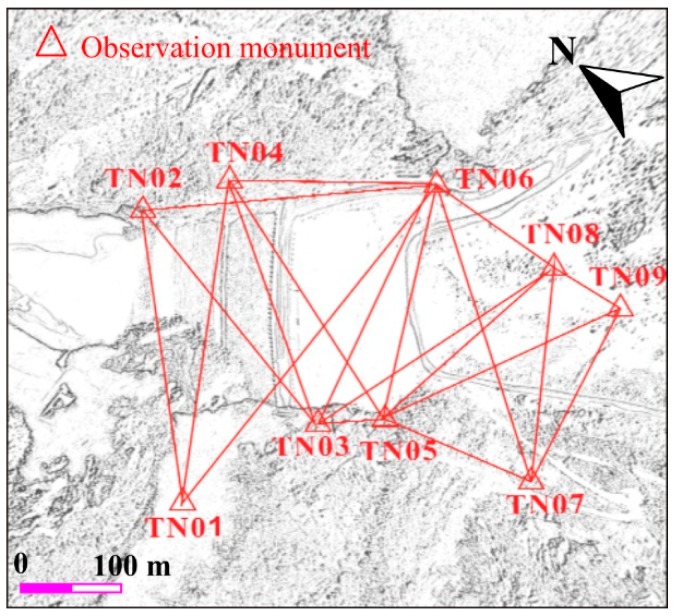
Layout of the geodetic network.

A geodetic network in the nearby of the earth-rock dam has been constructed since the commencement of the project in the year of 2011, which consists of nine control points materialized by observation monuments with a forced centering device. Of the nine control points, five are located on the left bank of the Dadu River, with other four on the right bank, and Figure 2 shows the layout of the geodetic network. The maximum and minimal side lengths of the network are about 1200 m and 210 m, respectively. Datum points TN03, TN04 and TN06 are equipped with inverted plumb line system. Thanks to the good conservation of these observation monuments, the geodetic network has been working well since the beginning. Two measurements of the geodetic network have been performed in October 2016, April 2017, respectively, by means of a Leica TM30 GeoRobot (provided by Leica Geosystems AG, Heerbrugg, Switzeland). The observation results of the TN04 and TN06 based on the inverted plumb line system shows the two points of the control net are stable. Thus, they are used as starting point for the classic free network adjustment calculation. The mean square error of points is less than +2 mm using the classic free network adjustment. Of the two measurements, the mean square error of the weakest point is 1.61 mm and 1.76 mm, respectively. And the average relative mean square error of the weakest side is 1/283,000 and 1/258,000, respectively. The results of the measurements and adjustment calculation indicate that the geodetic network is fine enough for the coordinate measurement of the scanner.

## 3. Methods

### 3.1. Terrestrial Laser Scanning

Terrestrial laser scanning is a quite new surveying method in geodesy. The scanner illuminates the target object with pulsed laser and records the returned pulse of the laser. It calculates the distance from itself to the target object surface automatically by timing the round-trip time of one pulse of the laser. Utilizing the time-of-flight (TOF) technology, the TLS is able to record dense point-clouds over an extremely short period of time. The modern laser scanner provided by the manufacturer shows a significant higher speed of data acquisition, compared to the conventional surveying instruments like total station [23]. Moreover, the TLS can also record the intensity of the reflected pulsed laser and RGB color data of the target object.

**Figure 3:**
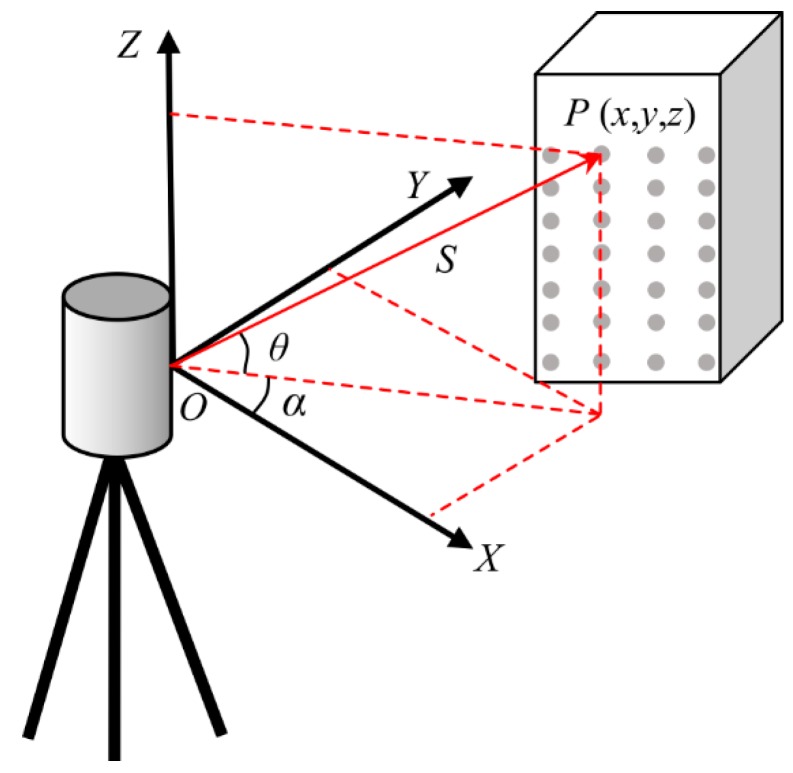
Measuring principle of the terrestrial laser scanning (TLS).

Let the origin of the Cartesian (rectangular) coordinate system coincide with the center of the laser scanner, where X and Y are two axes perpendicular to each other lying in the horizontal plane, and the Z axis is oriented upwards and perpendicular to the horizontal plane (Figure 3). Then, the coordinates of the laser point in the there-dimensional (3-D) space can be calculated from Equations (1) and (2) [24].

(1) S=c×(TOF2)

(2) $$\{\begin{array}{c} x=S\cdot \cos\theta \cdot \cos\alpha \\ y=S\cdot \cos\theta \cdot \sin\alpha \\ z=S\cdot \sin\theta \end{array}$$

where *c* is the speed of light; *TOF* is the time of flight of the laser pulse; *S* is the distance from the scanner to the reflecting surface; *θ* is the angle between the line *OP* and the *XY* plane; and α is the angle between *X* axis and the orthogonal projection of the *OP* onto the plane *XY*.

In this paper, a pulse-based scanner, Riegl VZ-400 (provided by RIEGL Laser Measurement Systems, Horn, Austria), is applied to survey the study area. The scanner is capable of measuring the distance ranging from 1.5 m to 600 m. Due to the echo digitization and online waveform processing technique, the highest angular resolution that this instrument can achieve is 0.0005°, and its efficient measurement rate is up to 120,000 points per second. Besides, it offers a wide field of view up to 100° vertical and 360° horizontal. As such, this equipment is suitable for the data acquisition in the vested condition.

### 3.2. Data Acquisitions and Preprocessing 

Measurement campaigns were performed after the filling of the dam. Due to the great size of the dam, it is impossible to acquire the point cloud data of the entire dam surface with only one scan. In addition, a relatively larger number of scans can increase the density of target point clouds, making sure that the overlap between different scans is enough for the alignment of different data sets.

Here, both the particular geologic of this case and the requirement of data processing, nine scans were performed at nine different stations distributed in the surrounding of the dam for the first time in October 2016, when the project began to impound. For each scan, the scanner was placed on the observation station. Then the coordinates of the scanner were measured based on the geodetic network. In this way, it was possible to get more precise coordinates of the scanner and reduce alignment error. It took approximately nine minutes to complete each 360° scan. The whole data set acquired in the first measurement campaign comprises one hundred and thirty-six million points in total, which is treated as the reference point cloud. The second measurement campaign was performed in April 2017 in a similar way as that in the first campaign, and both measurements were referred to the geodetic coordinate system.

These two sets of TLS data were preprocessed using the RISCAN PRO software. First, the geodetic coordinates of each scanner were extracted from the total station based on the geodetic network. By simply adding the instrument height, the geodetic coordinates of the scanner center were acquired. Then the geodetic coordinate data was input into the RISCAN PRO software for the raw scans by the backsighting orientation. In this way, the location of each scan center was determined. After the input of the coordinates, the orientation of each scan remained random. Therefore, a manual modification of the orientations was carried out to make the orientations in space correct. The second process was multi station adjustment. This process was performed using the iterative closet point (ICP) algorithm proposed by Besl and McKay [25]. In the first place scans were aligned pair by pair by means of lowering the “search distance” parameter from meters to some centimeters step by step. After a few adjustments, the alignment of the pair of scans led to an optimal rota-translation alignment matrix. Following this procedure a global alignment was employed for the whole scans to obtain a best fit alignment [26]. Both steps were based on the ICP algorithm and the latter step was executed for the purpose of distributing the residual registration error more homogeneously across the scans [25]. The global alignment of the nine scans resulted in the overall standard deviation of 0.0017 m, which meant the average distance between two closest points. The alignment error is mainly owing to the point spacing of the datasets in the overlap area and the point measurement error in practice. By increasing the point density in the overlap area and reducing the distance between the scanner and the overlap area, the standard deviation can be significantly lowered. In consideration of the high point density of the point clouds, this alignment error is primarily caused by point measurement error which can be reduced by fitting technique. Thus, the alignment error is acceptable for the deformation monitoring of the dam. Meanwhile, the four circular reflector targets were used as tie point for the calibration of the alignment. Similarly, the geodetic coordinates of its center points were measured by total station. Moreover, the geodetic coordinates of the center points were extracted from the scanning data. Then there were two sets of geodetic coordinates of the center points. By taking the geodetic coordinates acquired from the total station as datum, the mean square error of the four center points was calculated out. The mean square error of the points was no more than 0.0013 m, which proved that the alignment of the scans was successful. The alignment of the partial scans leaded to a single point cloud data set consisting of all scanned points.

**Figure 4:**
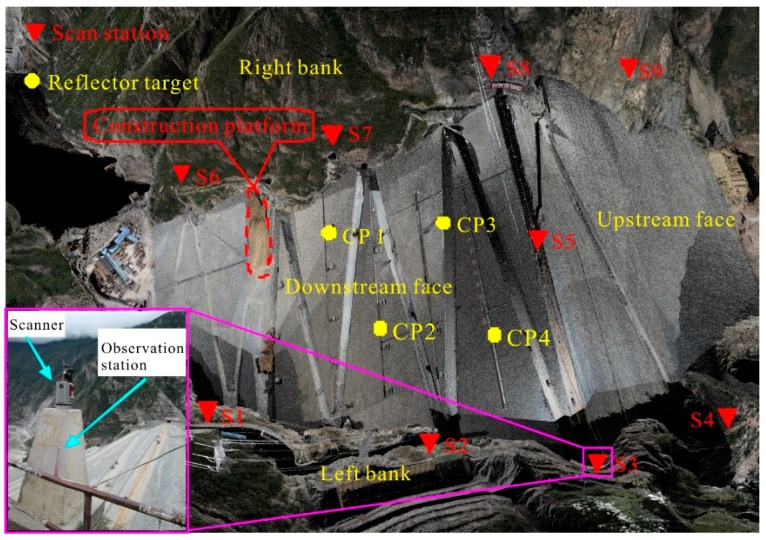
Aligned 3D model of the Changhheba dam (rendered view).

After alignment of all nine scans, there were some unneeded objects such as dust and vegetation in the point cloud. Via automatic and manual operation, the data was run through terrain filter to remove such points. Eventually, a single point cloud colored with RGB information obtained from the calibrated camera on top of the terrestrial laser scanner came into being, whose partial view is shown in Figure 4 below.

After the alignment of the point cloud, it comes to the stage of the triangulation. The basic input data that can be processed for NURBS surface reconstruction is the triangulated irregular network (TIN) rather than the point cloud. Hence, the TIN should be constructed based on the point cloud first, i.e., triangulation. The triangulation of points is also called “tessellation”. By using the Delaunay triangulation algorithm, each set of three closest points in the point cloud are connected to form a triangle, resulting in a non-overlapping triangulation as a whole. However, the initial triangulation generated automatically by the Delaunay triangulation algorithm usually has deficiencies, like acute angled triangles and holes. With automatic analysis, inconsistencies are detected and then repaired through automatic or manual operation. The final operation carried out to refine the TIN is the smoothing. The purpose of smoothing operation is to reduce the noise and therefore to ensure both the quality and accuracy of NURBS surface that will be constructed later. The downstream face of the Changheba dam is made of masonry; the dam surface is flat macroscopically. Thus, the smoothing operation is of central significance to the surface reconstruction.

**Figure 5:**
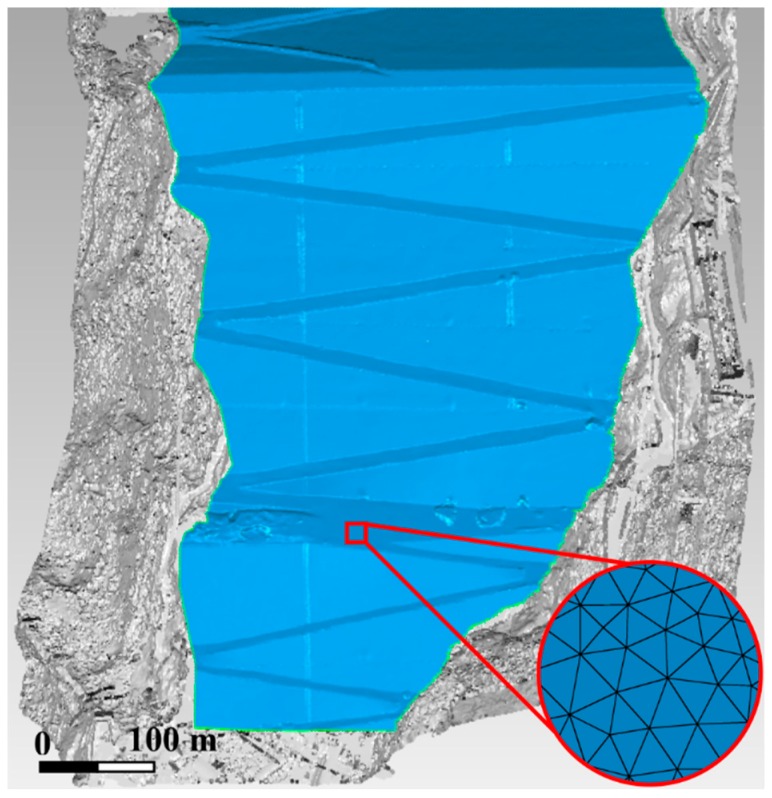
Triangulated irregular network (TIN) model of the Changheba dam.

The point clouds for the Changheba dam are converted into a polygon object with 3.48 billion triangles in total (Figure 5). Only points representing the dam surface are employed and converted into TIN, and other points beyond the survey area are not processed in this section.

The previously-constructed TIN is exported and then processed into a NURBS fitting surface model of the Changheba dam. With high accuracy and high spatial resolution, this NURBS surface model can be applied to detect the deformation of the dam surface for further study.

### 3.3. NURBS Modeling 

NURBS is the abbreviation of non-uniform rational B-splines, in which, the non-uniform means that the spacing of the knots is uneven, the Rational implies that the control point can be weighted, and the B-spline represents that B-spline is used as basis function. NURBS is a piecewise rational vector polynomial function advocated by Versprille in 1975 [27]. It can generate and represent arbitrary curves and surfaces better than other functions like radial basis function [28], which can be in either standard shapes or free-form shapes. The NURBS is widely used in computer graphics and the CAD/CAM industry, due to its great flexibility and precision. Besides, fitting surface is a useful means of reducing noise. Thus, NURBS is a useful tool to build surface model of natural land relief [29].

A NURBS surface *P*(*u, v*) = {*x*(*u, v*), *y*(*u, v*), *z*(*u, v*)} is a piecewise rational surface defined by Equation (3):

(3) $$P\left(u,v\right)=\frac{\sum_{i=0}^{m}\sum_{j=0}^{n}w_{i,j}N_{i,k}\left(u\right)N_{j,l}\left(v\right)d_{i,j}}{\sum_{i=0}^{m}\sum_{j=0}^{n}w_{i,j}N_{i,k}\left(u\right)N_{j,l}\left(v\right)}$$

where, the *d_i,j_* (*i* = 0, 1, …, m; *j* = 0, 1, …, n) are the control points representing a topological mesh, *w_i,j_* is the so-called weights, and the *N_i,k_*(*u*), *N_j,l_*(*v*) are the normalized B-spline basis functions defined on the non-periodic knot. For instance, the mathematical expression of the *N_i,k_*(*u*) can be defined recursively as follow,

(4) $$N_{i,0}\left(u\right)=\{\begin{array}{c} \begin{array}{cc} 1, & u_{i}\leq u\leq u_{i+1} \end{array}\\ \begin{array}{cc} 0, & otherwise \end{array} \end{array}$$

(5) $$N_{i,k}\left(u\right)=\frac{u-u_{i}}{u_{i+k}-u_{i}}N_{i,k-1}\left(u\right)+\frac{u_{i+k+1}-u}{u_{i+k+1}-u_{i+1}}N_{i+1,k-1}\left(u\right)$$

To generate a NURBS surface, three groups of parameters, control points *d_i,j_*, weight factors *w_i,j_* and the knot vectors ***U*** and ***V*** must be determined. On the grounds of the NURBS theory, the dam surface is constructed from the TIN following the four procedures: panel demarcation, surface patch insertion, grid generation, and NURBS surface construction. The four procedures are executed in sequence to construct the NURBS surface, as explained in the following.

**Figure 6:**
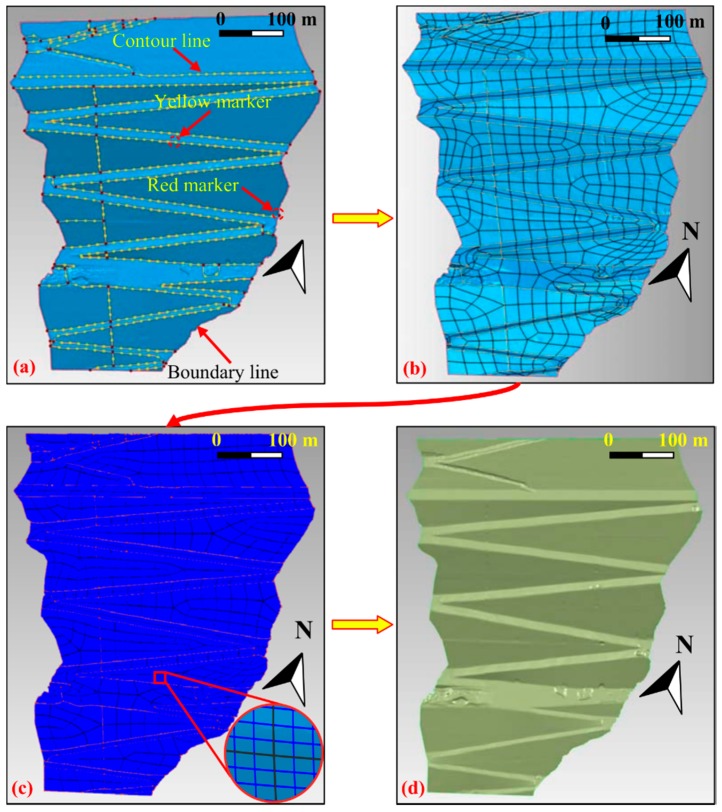
Non-uniform rational B-splines (NURBS) surface construction procedures: (**a**) panel demarcation; (**b**) surface patch insertion; (**c**) grid generation; (**d**) NURBS surface construction.

Data segmentation must be carried out to precisely construct the NURBS model of dam surface primarily. In this step, the characteristic lines are extracted from the TIN model. The surface curvature detection technique is adhibited for data segmentation. After setting the proper curvature level, the curvature detecting is performed and the contour lines are placed in the areas of curvature. In the meantime, Markers are highlighted on the contour lines. There are two different kinds of markers. A red marker indicates a corner, while a yellow marker represents a non-corner point of inflection. The contour lines and the boundary lines are used as panel demarcation lines dividing the TIN model into forty-three panels (Figure 6a). If a high curvature level was specified, fewer panels would be acquired. Then the precision the NURBS fitting surface would be affected. But more panels require more processing power and lower the operability. In this study, both the precision requirement and operability, the forty-three panels are proper for the NURBS fitting surface. The demarcated panels will be the containers for the surface patches in the next step. 

On the basis of the result of the panel demarcation, the patch boundary structure is generated, which means that four-sided patches are inserted into each panel (Figure 6b). A surface patch is a four-sided subdivision of a panel that is approximately equilateral. Based on the auto estimate technique, the target patch count is automatically calculated depending on the size and the smoothness of the panels. As the panels are very irregular and the patches are relatively regular, the adaptive insertion technique is applied in order to acquire fairly uniform patches, which contributes to the precision of the NURBSB model. Using the adaptive surface patch insertion technique, the shape of the surface patches is determined. The model consists of eight hundred and thirty-nine surface patches. From Figure 6b, it can be seen that the patches that comprise a panel differ in size, and the shapes of most patches are close to the rectangle. The inserted patches will be the containers for the grids in the following procedure.

After the surface patches are inserted, a further process is grid generation. A grid is a quadrangular mesh constructed in every patch. The mesh is made up of a forty-by-forty set of rectangles, which means that each panel consists of one thousand and six hundred grids. The quadrangular mesh density can be specified by the user. Needless to say, a finer grid produces greater precision in the eventual NURBS surface. The grid generation process creates an ordered u-v grid in every patch on the model (Figure 6c).

For the free-form surface of the dam, the exact NURBS surface can be ultimately generated on the TIN using the surface fitting technique. The NURBS surface of the dam is shown in Figure 6d. The consequent high accuracy and high spatial resolution surface model captures very well the morphology and geometry features of downstream face of the Changheba dam, which can be then inquired into the deformation characteristics of the dam [30,31].

### 3.4. Deformation Measurement by Shortest Distance (SD) Comparison

Deformation can be detected by making geometrical comparison between multi-temporal surface models. In this study, the shortest distance algorithm is applied to the deformation measurement. It is noted that the normal directions of the two surfaces should be generally consistent before the comparison. The algorithm can still work even when the surface normal vector is biased. For each point *i* (*x_i.tes_*, *y_i.tes_*, *z_i.tes_*)*^T^* in the test model, the algorithm searches for its nearest corresponding point *j* (*x_j.ref_*, *y_j.ref_*, *z_j.ref_*)*^T^* in the reference model and computes the SD vector, *V_i_*, that starts in point *i* and ends in point *j* (Equation (6)) [32].

(6) $$V_{i}={\left(\Delta x_{i},\Delta y_{i},\Delta z_{i}\right)}^{T}={\left(x_{i.tes},y_{i.tes},z_{i.tes}\right)}^{T}-{\left(x_{j.ref},y_{j.ref},z_{j.ref}\right)}^{T}$$

**Figure 7:**
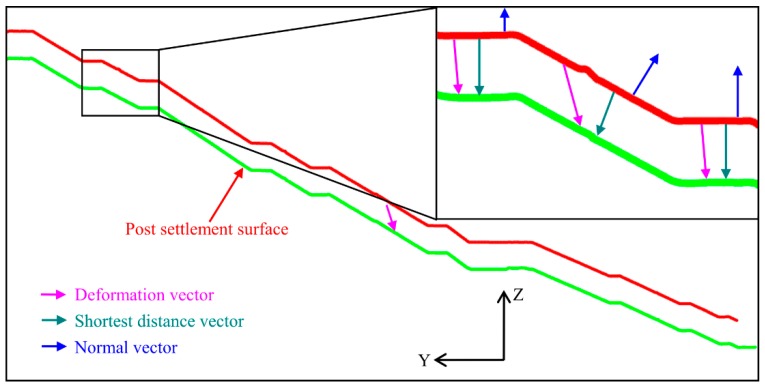
Conceptual comparison of the profiles.

For the NURBS fitting surface, the central points of grid are used to calculate the shortest distance. The calculated SD vectors do not necessarily represent the real displacements of the object. They are the shortest distances from the point in the test model to the nearest neighbor point in the reference model mathematically (Figure 7). Yet, the shortest distances (SDs) are useful for deformation measurement, since they allow for the detection of the vertical, horizontal, and oblique distances. In this paper, the sign conventions are defined as follow. Positive SDs indicated that the points in the test model are in front of or above those in the reference model. They may not appear to be continuous and can be interpreted as material stack owing to human activity. Negative SDs signified that the detecting part is behind or below the reference dataset, which are related to the vertical settlement and subsidence. 

To analyze the deformation of the structure, it is necessary to choose the proper coordinate system [33]. Herein, the main deformation of the dam surface taking place during the operation period is the settlement along the vertical direction. Hence, the Z direction is set as the vertical direction. And the Y direction is set in the direction parallel to the river. The X direction is set in the direction perpendicular to the river. Then, the shortest distance calculated from multi-temporal models can represent the deformation. The calculated data is useful for the dam deformation monitoring [32], with which the deformation of the downstream face can be analyzed in detail.

## 4. Results

### 4.1. Deformation Analysis

It is well known that the earth-rock dams are usually subjected to deformation over a relatively long time after the filling. Obviously, the deformation distribution is an essential indicator of the potential instability of the dam. Thus, periodic monitoring of the dam deformation during operation period is of great importance. The deformation of the surface is always larger than the interior due to the effect of displacement cumulation. Therefore, monitoring the surface deformation is an effective way of determining the serviceability of the dam. By introducing the TLS and NURBS technology, making comparison between different multi-temporal scans provides the plentiful and vital information for analysis of the deformation distribution of the dam.

For the Changheba dam studied herein, the NURBS model in October 2016 was treated as reference model, while the NURBS model in April 2017 was regarded as the test model. The reference model was subtracted from the test model. In this way, more than eight hundred thousand central points of grid in the test model were calculated and color-coded mappings of the differences were generated, showing the deformation distribution over the monitored time interval during the operation period.

**Figure 8:**
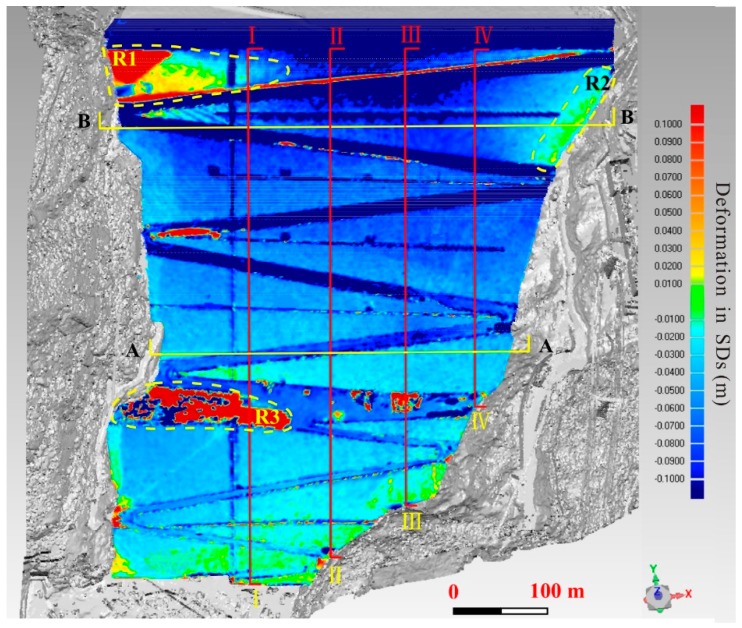
Deformation distribution of the Changheba dam. SDs: shortest distances.

The deformation distribution of the Changheba dam from October 2016 to April 2017 is shown in Figure 8. On account of the high water level in April 2017, the point cloud data of the upstream face was not obtained, at which the deformation cannot be calculated with only point cloud data in October 2016. As a result, only the deformation of the downstream face is presented. Negative changes representing the dam settlement are shown in cold colors. As shown, the dam has experienced settlements continuously after the filling, which can be interpreted as consolidation. The differential deformation is relatively significant. On top of the dam, the maximum deformation value is −0.0976 m, while the deformation values near the dam toe are close to zero. Along the stream direction, the deformation values of the dam surface get gradually smaller from the top to the toe. In the direction perpendicular to the river, the envelope of deformation exhibits a counter-arch shape (Figure 8). That is to say, the deformation in the middle of the dam is larger than that at the sides of the dam at the same elevation. The deformation of the zigzag road is much larger than its near regions due to the intense human activities.

There are three main regions with positive changes in Figure 8, marked as R1, R2, and R3. R3 was in the construct platform in elevation 1551 m in virtue of the construction activities. The other two parts R1 and R2 are located near two abutments. The point cloud data here is missing in the second measurement campaign in April 2017. The hole was filled manually in the process of NURBS model construction. Thus, the calculated result does not indicate the real changes happening in the site.

### 4.2. Deformation Mechanism

**Figure 9:**
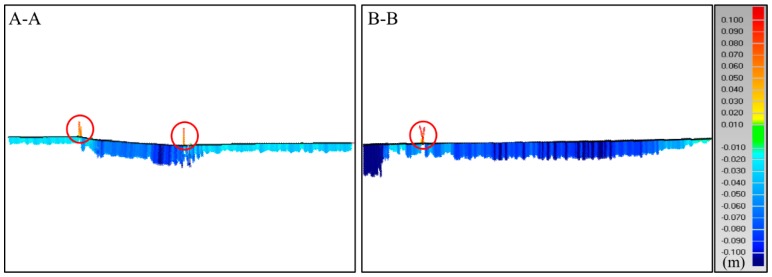
The deformation distribution along the two cross-sections.

Figure 9 shows some deformation distribution along the cross sections A-A, B-B (identified in Figure 8). As shown in Figure 8 and Figure 9, this dam continued to undergo deformation after the completion of filling of the dam, with the maximum deformation value up to virtually 90 mm in the two cross sections. In the meanwhile, the deformations in the middle at both cross-sections are normally larger than those near the banks, which caused by the effects of constraint from the banks on both sides. The envelopes of deformations of both cross sections are asymmetric, reflecting the fact that the earth-rock dam has been constructed in an asymmetric canyon.

It is worth mentioning that the deformations in cross section A-A are much smaller compared to those in cross section B-B. One of the reasons is that the lower part of the dam has been subjected to settlement over a relatively longer time than the upper part of the dam. The lower part of dam started to deform right after its filling, at this time the upper part has not been filled. Another reason is that, after filling of the upper part, additional settlement of the lower part will be induced due to the addition of gravity load on the top. Thus, the relative smaller deformation of lower part is the consequence of combination of a longer period of consolidation and higher stress conditions.

**Figure 10:**
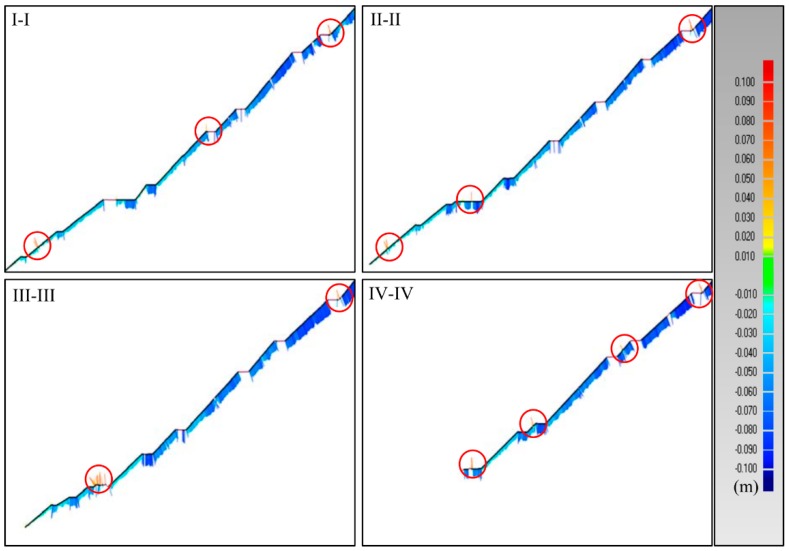
The deformation distribution along longitudinal sections.

Figure 10 show some deformation distribution along the longitudinal sections I-I, II-II, III-III, and IV-IV, as marked in Figure 8. The magnitudes of deformations increase nonlinearly with the increasing elevation. The relatively larger deformations of the upper part demonstrate that the inner part of the dam has experienced a rapid consolidation during the investigation period. It can also been seen that the deformations of longitudinal sections II-II, III-III are always larger than those at the other two sections I-I, IV-IV, at the same elevation, which means the deformations in the middle are generally bigger than those on the sides at the same elevation. This corresponds with the conclusion drawn from Figure 9. In the meanwhile, there exist several abnormal regions circled in red in Figure 10, which represent the positive changes taking place on the dam surface. This phenomenon results from the human activities and the data missing.

## 5. Discussions

**Figure 11:**
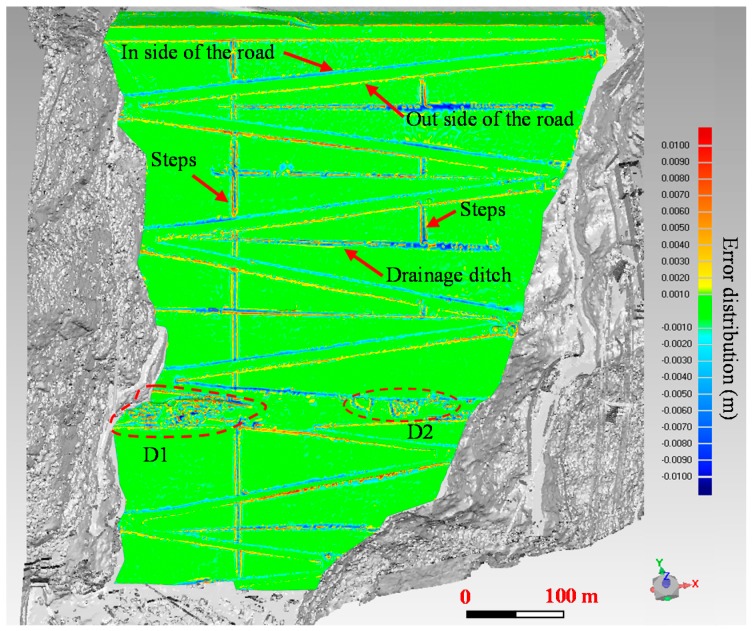
Error distribution in the NURBS modeling process.

In this paper, a new deformation monitoring method for large structure is formulated. The field experiments are carried out to test its feasibility. In practical works, reducing error is of vital importance for deformation monitoring. According to the different stages of the methodology, the possible errors can be divided into three parts: instrument error, alignment error and modeling error. Many researches have been done on the instrument error and alignment error for the past decades [34,35]. Here, the modeling error of the NURBS technology is analyzed. The NURBS surface model constructed is a kind of fitting surface. There exist some errors in the constructed surfaces, and the error distribution is shown in Figure 11 by making comparison between the original point cloud and the NURBS fitting surface. The overall error of the fitting surface is relatively small, and the distribution of the errors is quite uniform. Errors in most parts are within ±0.002 m. The relatively larger errors are concentrated mainly in the places with large curvatures. One example is in the area of the construction platform including D1 and D2. Since the construction platform is not the focus of this current study, where the NURBS fitting surface is constructed roughly in order to reduce the size of the NURBS surface file. Nevertheless, the fitting surface may not be subtle enough to represent surface of objects with the large curvatures where large errors are likely to be induced. Another area with relatively large errors is at the two sides of the zigzag road form dam crest to dam toe. The NURBS surface is about 0.006 m higher than the original points inside the zigzag road. On the contrary, the NURBS surface is approximately 0.006 m lower than the original points outside the zigzag road. This is also due to the large curvatures of the steps and the drainage ditch. As the larger errors don’t exist in the main study reaches, the constructed NURBS surface can be applied to the analysis of the deformation distribution. Error analysis above proves that the NURBS fitting surface is useful way of surface reconstruction.

## 6. Conclusions

The deformation distribution has a significant influence on the stability and safety of large artificial and natural structures. By taking full advantage of the TLS and NURBS technology, a new methodology of deformation monitoring and analysis is presented. Using the earth-rock dam as an example, the point cloud gained by TLS allows the detection of small deformation for its high accuracy and capability of target acquisition. The NURBS model based on the point cloud is characterized by high precision and high spatial resolution. Eventually, the holistic deformation distribution of the downstream face is shown in the cloud chart. The deformation monitoring achieved great success with the proposed methodology. The TLS has proved to be a valid solution for the deformation acquisition in three-dimensional space. The NURBS modeling technology is capable of dealing with a huge number of points and making use of them. In comparison with the traditional monitoring, the methodology that integrates TLS and NURBS technologies permits a better grasp of the deformation distribution of the large structures.

The millimeter level measurement requires that the data acquisition is performed with great patience and plenty of time. In the future works, data acquisition in the field should be optimized to reduce the field working time. Besides, the data processing is also time-consuming and complicated. Several procedures require personal experience and expertise. These are several potential areas in terms of improving the performance of the presented methodology, in which further research will be conducted.
